# The role of left insula in executive set-switching: Lesion evidence from an acute stroke cohort

**DOI:** 10.1016/j.cortex.2017.11.009

**Published:** 2018-10

**Authors:** Andreja Varjačić, Dante Mantini, Jacob Levenstein, Elitsa D. Slavkova, Nele Demeyere, Céline R. Gillebert

**Affiliations:** a Department of Experimental Psychology, University of Oxford, Oxford, United Kingdom; b Department of Brain and Cognition, University of Leuven, Leuven, Belgium; c Department of Health Sciences and Technology, ETH Zurich, Zurich, Switzerland

**Keywords:** Executive functions, Trail Making Test, Stroke, Set-switching, Lesion-symptom mapping

## Abstract

Impairments in executive functions are common in stroke survivors, both in the acute and in the chronic phase. However, little is known about the underlying lesion neuroanatomy of these deficits. This study aimed to elucidate the pattern of brain damage underlying executive dysfunction in a large and acute stroke cohort. Executive set-switching deficits were evaluated by a shape-based analogue of the Trail Making Test (from the Oxford Cognitive Screen) in a consecutive sample of 144 stroke patients (age: 70 ± 15 years, examination: 5 ± 4 days post-stroke; brain imaging: 1.7 ± 2.9 days post-stroke). A voxelwise lesion-symptom mapping analysis was performed by combining executive set-switching accuracy scores with manually delineated lesions on computerized tomography or magnetic resonance imaging scans. The analysis showed that lesions within the left insular cortex and adjacent white matter predicted poorer executive set-switching. Further analyses confirmed that the lesion effect in the left insula survived correction for the low-level visuospatial and motor component processes of executive set-switching. In conclusion, the study provides lesion-based evidence for the role of the left insular cortex in flexible switching of attention. The findings are consistent with emergent models of insular function postulating the role of this region in regulatory aspects of goal-directed behaviour.

## 1 Introduction

Flexible switching of attention between tasks, operations and stimulus sets reflects a core aspect of executive control (Miyake et al., 2000). Deficits in executive set-switching have been documented in both acute (Tamez et al., 2011) and chronic stroke cohorts (Chan et al., 2015, Yochim et al., 2007). Mounting evidence highlights the importance of studying executive deficits that accompany stroke. For instance, a modulatory relationship between domain-general executive control mechanisms and domain-specific cognitive tasks has been observed within language (Brownsett et al., 2014) and visuo-spatial attention domains (e.g., Robertson et al., 1997, Singh-Curry and Husain, 2009). Further, the presence of an early executive impairment may predispose stroke survivors for experiencing reduced quality of life in the chronic phase (Nys et al., 2006).

A commonly used instrument for mapping executive deficits in neurological populations is the Trail Making Test (TMT), a visuo-motor search task that induces switching between competing stimulus sets (e.g., Sánchez-Cubillo et al., 2009). Due to its purported executive demands (e.g., Arbuthnott and Frank, 2000, Kortte et al., 2002), early lesion research speculated that the TMT may be used as a tool for detecting executive impairment stemming from frontal lesions (Stuss et al., 2001). However, several recent studies did not support the specific role of the frontal areas in mediating set-switching in the TMT, both in (sub)acute (<3 months; Tamez et al., 2011, Muir et al., 2015) and chronic phases post-stroke (>3 months, Chan et al., 2015, Muir et al., 2015). Specifically, these studies failed to demonstrate that patient categorisation, either into frontal versus non-frontal groups (Chan et al., 2015, Tamez et al., 2011), or based on the stroke involvement with the nodes of a predefined “executive network” (Muir et al., 2015), discriminated between the TMT-derived indices of set-switching.

By contrast, studies utilising a more sensitive approach of categorising patients based on the presence of a lesion in a voxel-wise fashion (see Bates et al., 2003, Rorden et al., 2007), suggest the involvement of diverse frontal regions in TMT performance. For instance, a large sample study of chronic brain-injured patients (N = 236) found that lesions within the rostral anterior cingulate cortex predicted less efficient set-switching performance (Gläscher et al., 2012). Another large-sample study of individuals with penetrating head injuries (N = 182) reported an association between regionally non-specific lesions within the left prefrontal cortex, anterior cingulate, insula, parietal and temporal areas and lower executive functioning, as evaluated by the Delis-Kaplan Executive Function System (D-KEFS) tests that included the TMT (Barbey et al., 2012). Furthermore, damage to the left dorsomedial prefrontal cortex was associated with slower set-switching in a study of 27 frontal chronic brain-injured patients with heterogeneous aetiologies (Miskin et al., 2016). Finally, lesions within the right dorsolateral prefrontal cortex predicted higher incidence of set-switching errors in a sample of 30 acute, right-hemispheric and predominantly frontal stroke patients (Kopp et al., 2015). Although these voxel-lesion-symptom mapping (VLSM) studies collectively suggest that frontal areas are important for executive set-switching, the regionally specific frontal contributions remain inconclusive. In addition, the use of chronic brain-injured samples or small sample sizes in these studies makes it difficult to tease apart the contributions of localised brain damage from the long-term spontaneous plasticity effects (see Gillebert and Mantini, 2013, Guerra-Carrillo et al., 2014, Pascual-Leone et al., 2005).

Neuroimaging studies of the TMT performed in healthy volunteers suggest that both frontal and non-frontal areas are important for executive set-switching. Specifically, functional magnetic resonance imaging (fMRI) adaptations of the TMT contrasted the set-switching to a control condition and revealed brain activations in the prefrontal cortex, including the left lateral prefrontal cortex (Moll, de Oliveira-Souza, Moll, Bramati, & Andreiuolo, 2002), left superior frontal gyri (Zakzanis, Mraz, & Graham, 2005) and right-lateralised ventro-lateral prefrontal cortex (Jacobson, Blanchard, Connolly, Cannon, & Garavan, 2011). Further, less consistent non-frontal contributions to the set-switching component of the TMT were also observed across these studies, including insular, parietal and temporal activations. In line with this, fMRI studies that utilised established switching paradigms, including task-switching (Dreher, Koechlin, Ali, & Grafman, 2002), stimulus-response reversal (Dove, Pollmann, Schubert, Wiggins, & von Cramon, 2000), and the Wisconsin Card Sorting Task (Monchi, Petrides, Petre, Worsley & Dagher, 2004), implicated a widely distributed circuitry of both frontal and non-frontal regions in executive set-switching. Indeed, two meta-analyses of set-switching reported converging evidence that in healthy individuals, set-switching operations engage a widespread neural circuitry comprising superior parietal, premotor and anterior insular regions in addition to consistently reported prefrontal activation effects (Derrfuss et al., 2005, Wager et al., 2004).

Overall, although neuroimaging findings suggest that executive set-switching performance is mediated by a circuitry that extends beyond prefrontal cortex, a high regional variability of the reported effects makes it difficult to synthesise the findings across the studies. In addition, while VLSM studies highlight the involvement of the prefrontal cortex in executive set-switching in chronic brain-injured cohorts (Barbey et al., 2012, Gläscher et al., 2012, Miskin et al., 2016), lesion-mapping studies of executive deficits in acute stroke patients are lacking. The only identified VLSM study of executive set-switching in an acute stroke cohort used a restrictive sampling criteria based on the anatomical lesion location and included 30 patients (Kopp et al., 2015).

The current study aimed to elucidate the neuroanatomical underpinnings of executive set-switching deficits in a large (N = 144), consecutive, and acute stroke sample. Executive set-switching performance was quantified by a shape-based TMT analogue included in the Oxford Cognitive Screen (OCS) (Demeyere, Riddoch, Slavkova, Bickerton, & Humphreys, 2015), a stroke-specific screening tool covering the domains of attention and executive function, language, memory, praxis and number processing. In contrast to the standard TMT, which requires number-letter switching, the shape-based TMT analogue requires alternation between task-relevant shapes in order of size. The shape-based TMT analogue is intended to provide a more sensitive screen of executive deficits in stroke, as it permits assessing patients who are impaired in language and/or numerical sequencing (Demeyere et al., 2015). We used a voxel-wise approach (Bates et al., 2003) to map acute lesion data (mean stroke to scan interval = 2 days) onto acutely evaluated executive set-switching deficits (mean stroke to test interval = 5 days). The use of an acute stroke sample allowed the interrogation of lesion-deficit coupling in the absence of confounding long-term spontaneous plasticity effects (e.g., Gillebert and Mantini, 2013, Guerra-Carrillo et al., 2014, Pascual-Leone et al., 2005). Indeed, there is evidence suggesting a more reliable infarct-deficit mapping for acute, compared to chronic, behavioural datasets (Ochfeld et al., 2010).

## 2 Material and methods

### 2.1 Participants

The study cohort included 144 acute stroke patients (62 females; mean age = 71 ± 15 years) recruited consecutively from the acute stroke unit at the John Radcliffe Hospital, Oxford. This study involved the collection of acute behavioural data (mean stroke to test interval = 4.9 ± 3.8 days; with 73% of cases tested within the first week following hospital admission) and acute clinically obtained brain imaging scans (mean stroke to scan interval = 1.7 ± 2.9 days; Table 1). Neuropsychological examination was performed on the acute ward by means of the OCS.

**Table 1:** Demographic, neuroimaging and stroke data.

	N	Mean	SD
Age (years)	143	70.68	14.61
Education (years)	91	11.86	2.78
Gender (M/F)	144	82/62	N/A
Handedness (R/L/A)	117	99/16/2	N/A
Aetiology (Ischemic/Haemorrhagic)	144	113/31	N/A
Lesion side (R/L/B)	144	47/60/37	N/A
Lesion size (voxels)	144	4478	7004
Modality (CT/MRI)	144	120/24	N/A
OCS version (A/B)	144	107/37	N/A
Stroke to test interval (days)	140	4.93	3.80
Stroke to test interval (weeks) (1st/2nd/3rd)	140	105/29/6	N/A
Stroke to scan interval (days)	129	1.70	2.92

Inclusion criteria for the study were as follows: (i) patients were within 3 weeks of a confirmed diagnosis to have had an ischemic or haemorrhagic stroke; (ii) a record of both behavioural and brain imaging data acquired within 3 weeks post-stroke was available; (iii) brain imaging scans were of high enough quality to allow accurate manual tracing of stroke lesions; specifically, there were no imaging artefacts on the computerized tomography (CT) or magnetic resonance imaging (MRI) scans and no accompanying major structural brain abnormalities (e.g., severe cortical atrophy, small vessels disease etc.) were detected; (iv) patients presented no symptoms of egocentric hemi-spatial visual neglect, as assessed by the Hearts Cancellation task, included in the OCS (Demeyere et al., 2015), (v) patients had no current or previous diagnosis of psychiatric illness, degenerative disease, epilepsy, alcohol and/or drug abuse.

Demographics data and stroke information were obtained from the medical notes. Table 1 summarises demographic and stroke variables for the cohort. Written or witnessed informed consent was obtained from all participants. The study was approved by the UK National Research Ethics Service (Reference: 11/WM/0299).

### 2.2 Shape-based Trail Making Test Analogue from the Oxford Cognitive Screen

#### 2.2.1 Task and procedure

**Fig. 1:**
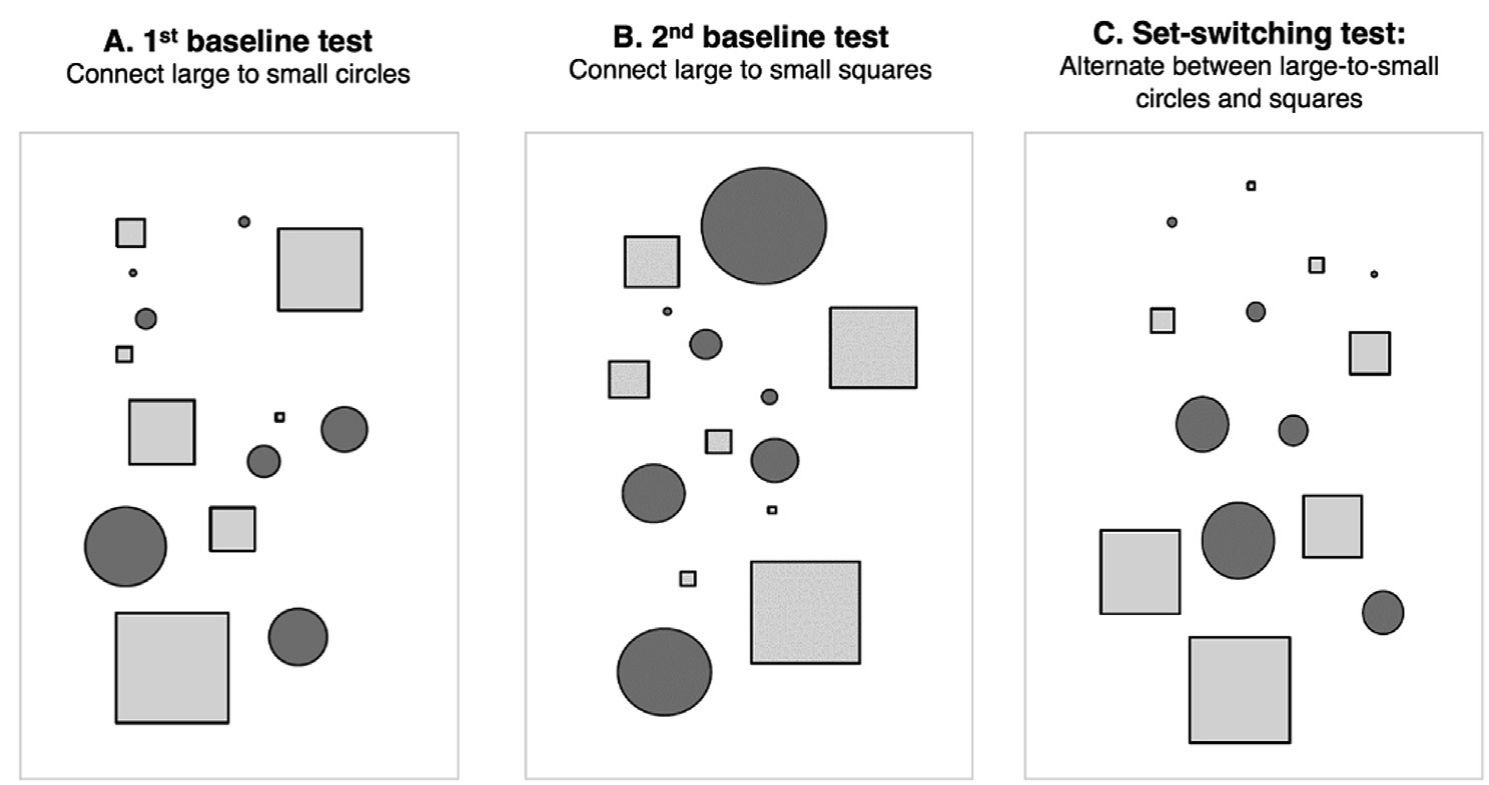
Stimulus sets for the shape-based TMT analogue from the OCS. A. First baseline test: participants connected large to small circles in the presence of square distractors. B. Second baseline test: participants connected large to small squares in the presence of circle distractors. C. Set-switching test: participants connected both circles and squares in descending and alternating order. Executive set-switching performance was expressed by the raw accuracy scores in the set-switching test.

Fig. 1 displays the stimulus sets for the shape-based TMT analogue included in the OCS. The use of shape-based stimuli, instead of letters and numbers, was intended to increase the task sensitivity for the detection of executive set-switching deficits in stroke by minimising language and numerical processing demands (Demeyere et al., 2015). The shape-based TMT analogue is thus an inclusive tool for the assessment of executive dysfunction independently of verbal and numerical disturbances. Accordingly, the hemispheric distribution of lesions in the current sample was not biased towards the inclusion of patients who were less likely to show verbal impairment, i.e., patients with right-hemispheric lesions. Specifically, of the entire sample of 144 patients, 47 patients had right-hemispheric lesions, 60 patients had left-hemispheric lesions and 37 patients had bilateral lesions (Table 1).

All participants were instructed to draw a line joining shapes in decreasing order of size on an A4 worksheet. In the shape-based TMT analogue, baseline performance was assessed across two tests. The first baseline test required participants to connect large to small circles in the presence of square distractors (Fig. 1A), whereas the second baseline test required connecting large to small squares in the presence of circle distractors (Fig. 1B). In the third set-switching test, participants were asked to connect both shape types in descending and alternating order (i.e., set-switching test; Fig. 1C). A short practice was administered before the start of each test to ensure that participants had an accurate comprehension of the task. Two versions of the shape-based TMT analogue (OCS-version A and OCS-version B) that differed in the shape type deployed (i.e., the use of triangles instead of squares–though no changes were made to the locations of the shapes) were administered between subjects. In contrast to the standard TMT, participants could take as much time as they needed to complete the baseline and set-switching tests, and performance speed was not emphasised by the experimental instruction. The self-paced nature of the task ensured that reduced performance accuracy, if present, was due to connecting fewer shapes in the incorrect order as a result of executive function deficits and not due to the fixed time constraint or the requirement to implement an additional task set (i.e., connect shapes as *fast* as possible).

#### 2.2.2 Behavioural analysis

Executive set-switching performance was expressed as the number of accurately connected shapes in the set-switching test of the shape-based TMT analogue (range of scores: 0–13). These raw accuracy scores were used as a dependent variable in the primary lesion-mapping analysis. Across two baseline tests, performance was expressed as the number of accurately connected shapes along a single stimulus dimension (range of scores: 0–6). The raw accuracy scores from these baseline tests were averaged for each participant and used as a covariate of no interest in the control lesion-mapping analysis. Due to the self-paced nature of the task, the completion time was highly variable across participants and was not correlated with the set-switching accuracy (r = .06, *p* = .48).

Prior to the lesion-mapping analysis, a series of tests were conducted to inspect whether set-switching accuracy scores were matched for each demographic and stroke variable. Specifically, the statistical tests included: Pearson's correlations (age, years of education, lesion size, stroke to test and stroke to scan interval), two-sample *t*-tests (gender, OCS version, imaging modality and stroke aetiology) and one-way ANOVAs (handedness and lesion hemisphere). We used a statistical threshold of *p* < .05, Bonferroni-corrected for multiple comparisons (uncorrected *p* < .004).

### 2.3 Lesion mapping

CT/MRI scans were acquired for all patients as part of their routine clinical assessment following stroke at the John Radcliffe Hospital, Oxford. A total of 120 axial scans in the CT modality (29–62 slices, slice thickness: 3–5 mm) and 24 scans in the MRI modality (24–192 slices, slice thickness: 1–6 mm) were acquired containing full-brain coverage. Out of 24 MRI scans, 2 axial scans were acquired using a T1-weighted sequence, 20 axial scans using a T2-weighted sequence and 2 coronal scans using a Fluid-attenuated inversion recovery (FLAIR) sequence.

#### 2.3.1 Image processing

Prior to delineating lesions, images were reoriented to the anterior commissure using Statistical Parametric Mapping 8 (SPM8) (Wellcome Trust Centre for Neuroimaging, London, United Kingdom). The lesion boundaries were manually delineated directly on the CT or the MRI image, slice-by-slice, in the plane of the highest resolution, using MRIcron (McCausland Center for Brain Imaging, Columbia, SC, USA). Manual delineation of lesions was performed by two independent experienced raters blind to the behavioural results. All lesion masks were smoothed at 5 mm full width at half maximum in the z-direction and binarised using a .5 threshold. Both the patients' clinical scans and delineated lesion masks were warped into 2 × 2 × 2 mm stereotaxic space using the Clinical Toolbox, based on SPM8 (Rorden, Bonilha, Fridriksson, Bender, & Karnath, 2012) (https://www.nitrc.org/projects/clinicaltbx/). The normalization algorithm in this toolbox involves affine (i.e., linear) and non-linear transformations, and uses cost-function masking (Brett, Leff, Rorden, & Ashburner, 2001). This procedure minimises the bias induced by the presence of abnormal areas by removing the lesion from the normalisation transforms (Brett et al., 2001). The Clinical Toolbox has the further advantage that it uses spatially-matched CT and MRI normalisation templates representative of a healthy elderly population (Rorden et al., 2012). The quality of normalisation was evaluated through visual inspection and was deemed satisfactory in all patients (see Figure S1 for an example of spatial normalization).

#### 2.3.2 Lesion-mapping analysis

We performed a VLSM analysis (Bates et al., 2003) to identify the voxels with a significant difference in the behavioural scores depending on the voxel lesion status (lesioned/intact). Specifically, we used a parametric VLSM tool that by default calculates and uses the lesion size of each patient as a covariate of no interest, and that allows one to add multiple covariates of no interest (https://langneurosci.mc.vanderbilt.edu/resources/). Correction for multiple comparisons was estimated using permutation-based thresholding (5000 iterations). Unless mentioned otherwise, a minimum of 10 patients with a lesion at any given voxel was necessary for the statistical test to be performed (e.g., Shahid et al., 2017), and the reported results survived a voxelwise threshold of *p* < *.001,* corrected for multiple comparisons based on cluster size and the permutation method (Kimberg, Coslett, & Schwartz, 2007).

The primary VLSM analysis was conducted on the raw accuracy scores from the set-switching test. The analysis was performed in the full sample of patients (N = 144), and repeated in the subset of patients with unilateral lesions (N = 107) and in the subset of right-handed patients (N = 99).

In a first set of control analyses, we corrected for the low-level visuo-spatial components of the set-switching test by using the average accuracy across two baseline tests as a covariate of no interest. Since the performance on the baseline tests was close to ceiling (Table 2), we repeated the VLSM analysis on the “executive score”, a composite score for executive functioning. This metric was computed by subtracting the set-switching accuracy (maximum score = 13) from the summed accuracy scores on the two baseline tests (maximum score = 12) (see Demeyere et al., 2015). The executive score thus reflects the change in the executive set-switching accuracy relative to baseline, with higher scores indicating lower executive set-switching ability.

**Table 2:** Accuracy data for the baseline and set-switching tests of the shape-based TMT analogue.

	M	SD	Min	Max
First baseline test	5.08	1.48	0	6
Second baseline test	5.34	1.22	1	6
Set-switching test	9.06	3.90	0	13

A second control analysis was performed to demonstrate the task specificity of the observed findings. To this end, we categorised the stroke patients depending on their lesion overlap with the identified clusters according to a predefined threshold (10%). We then evaluated the performance of both patient groups on the imitation task from the OCS (praxis domain). This mirror task requires participants to imitate meaningless hand and finger gestures performed by the examiner (Demeyere et al., 2015). The task was chosen for its similarity to the set-switching test in terms of difficulty level in healthy individuals (median score on the Imitation task 11; median score on the set-switching test: 12), the range of scores (Imitation task 0–12; set-switching test: 0–13), and the cut-off value for impairment (Imitation task 8; set-switching test: 7) (Demeyere et al., 2015). To evaluate the cross-task specificity of the findings, the scores on the set-switching test and the imitation task were re-scaled onto a 0–10 scale and analysed using a mixed ANOVA with lesion location (overlap/no overlap) as between-subject factor and task (set-switching test, imitation task) as within-subject factor.

## 3 Results

### 3.1 Behavioural data

Accuracy for the baseline and set-switching tests from the shape-based TMT analogue are summarised in Table 2. Of the entire sample (N = 144), 28.5% (N = 41) showed executive set-switching deficits (based on the evaluation of the set-switching accuracy scores against the normative cut-off of 7; Demeyere et al., 2015).

A series of statistical tests was performed to evaluate whether set-switching accuracy scores were matched for each demographic and stroke variable. A significant negative correlation between set-switching accuracy scores and lesion size was identified (r = −.28, *p* = .001). No significant relationship was detected between set-switching accuracy and age (r = −.03, *p* = .73), years of education (r = −.02, *p* = .86), stroke to test interval (r = −.13, *p* = .14) and stroke to scan interval (r = −.10, *p* = .27). Further, no significant differences in the set-switching accuracy scores were detected for gender (t(142) = .81, *p* = .42), handedness (F(2,114) = 1.14, *p* = .33), OCS version (t(142) = .06, *p* = .95), imaging modality (t(28.41) = .73, *p* = .47), stroke aetiology (t(142) = 1.15, *p* = .24) and lesion hemisphere (F(2,141) = 1.96, *p* = .15).

### 3.2 Lesion neuroanatomy

**Fig. 2:**
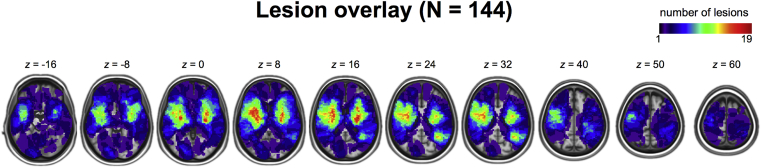
Overlay of the lesions of the 144 patients included in the VLSM analysis in stereotaxic space. The colour bar indicates the number of patients with lesions at each voxel. Images are displayed in neurological convention.

Fig. 2 shows an overlay of 144 lesion masks mapped into stereotaxic space. In line with empirical reports documenting predominantly subcortical lesion neuroanatomy in clinical stroke samples (e.g., Corbetta et al., 2015), the highest proportion of lesions involved damage to subcortical and insular areas.

#### 3.2.1 Primary VLSM analysis

**Fig. 3:**
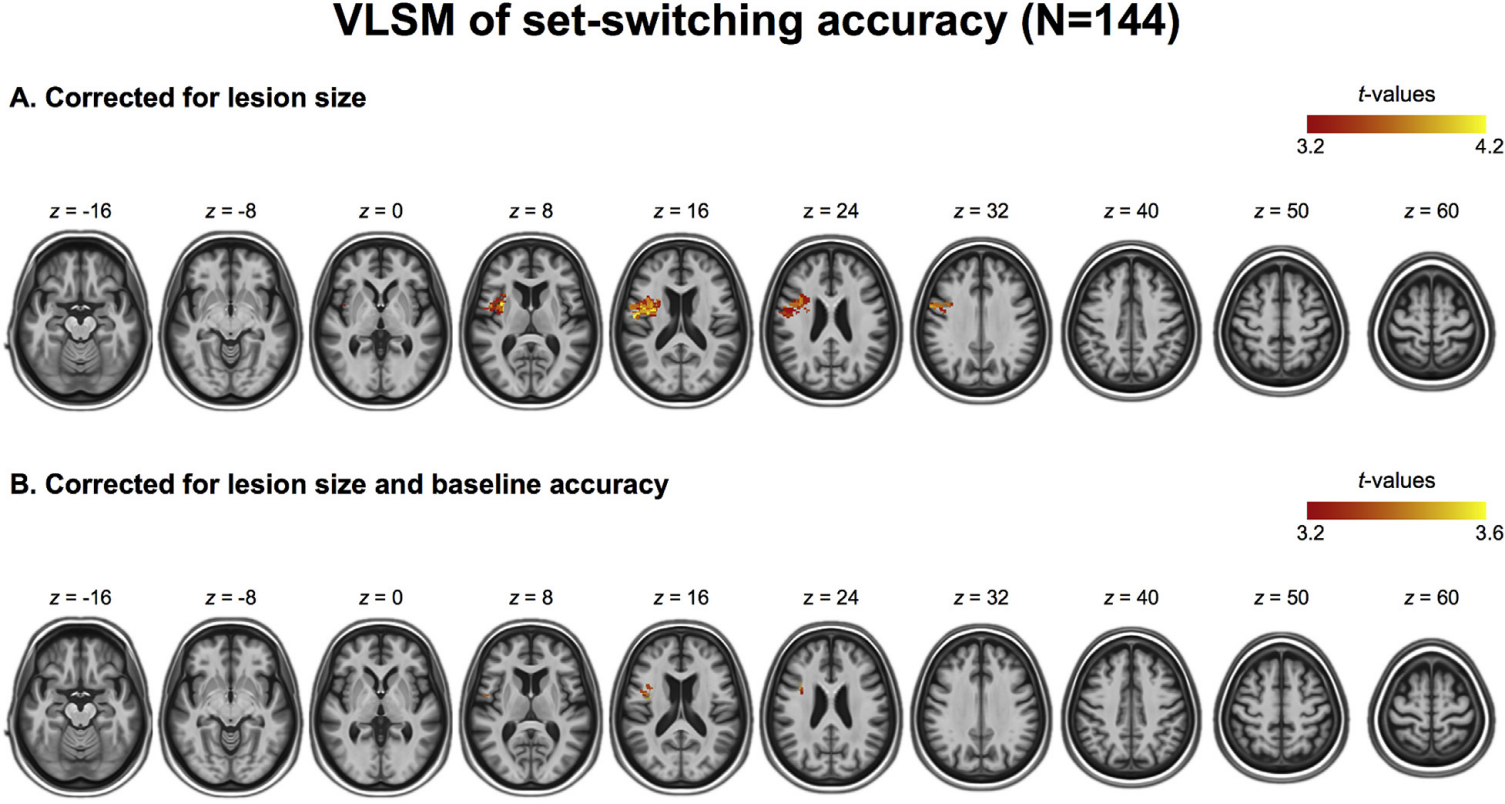
Results of the voxel-wise lesion symptom mapping (VLSM) analyses mapped onto the age-specific CT template. A. Lesion locations associated with lower set-switching accuracy scores identified in the primary VLSM analysis, with lesion size as a covariate of no interest. B. Lesion locations associated with lower set-switching accuracy scores identified in a control VLSM analysis, correcting for lesion size and low-level visuospatial and motor components of the baseline tests. Images are displayed in neurological convention.

To identify which brain areas, when lesioned, predict poor executive set-switching, accuracy scores from the set-switching test were submitted to a VLSM analysis with lesion size as a covariate of no interest. This analysis identified a cluster in the left insular cortex extending into precentral gyrus (1382 voxels, 2 × 2 × 2 mm^3^, centre of mass Montreal Neurological Institute (MNI) coordinates: x = −42, y = 0, z = 19) (Fig. 3A). The VLSM analysis of set-switching accuracy scores in patients with unilateral lesions (N = 107) identified a similar left-lateralised cluster in the insular cortex extending into the precentral gyrus (Figure S2A). Further, the lesion effect in the left insular cortex was robust to handedness, as indicated by the results of the VLSM analysis conducted in the right-handed patients only (N = 99) (Figure S2B).

#### 3.2.2 Analyses controlling for low-level visuospatial and motor demands

A secondary VLSM analysis was performed to correct the performance on the set-switching test for the low-level visuo-spatial and motor demands as measured by the two baseline tests. A VLSM analysis with accuracy on the baseline tests as covariate of no interest identified an overlapping cluster in the left insula extending into precentral gyrus (179 voxels, 2 × 2 × 2 mm^3^, centre of mass MNI coordinates: x = −41, y = 1, z = 17) (Fig. 3B). Similarly, a VLSM analysis on the executive score (an index of the set-switching accuracy relative to baseline) (Demeyere et al., 2015) showed an association between the left insula and poor executive set-switching (Figure S3).

#### 3.2.3 Analyses assessing cross-task specificity

**Fig. 4:**
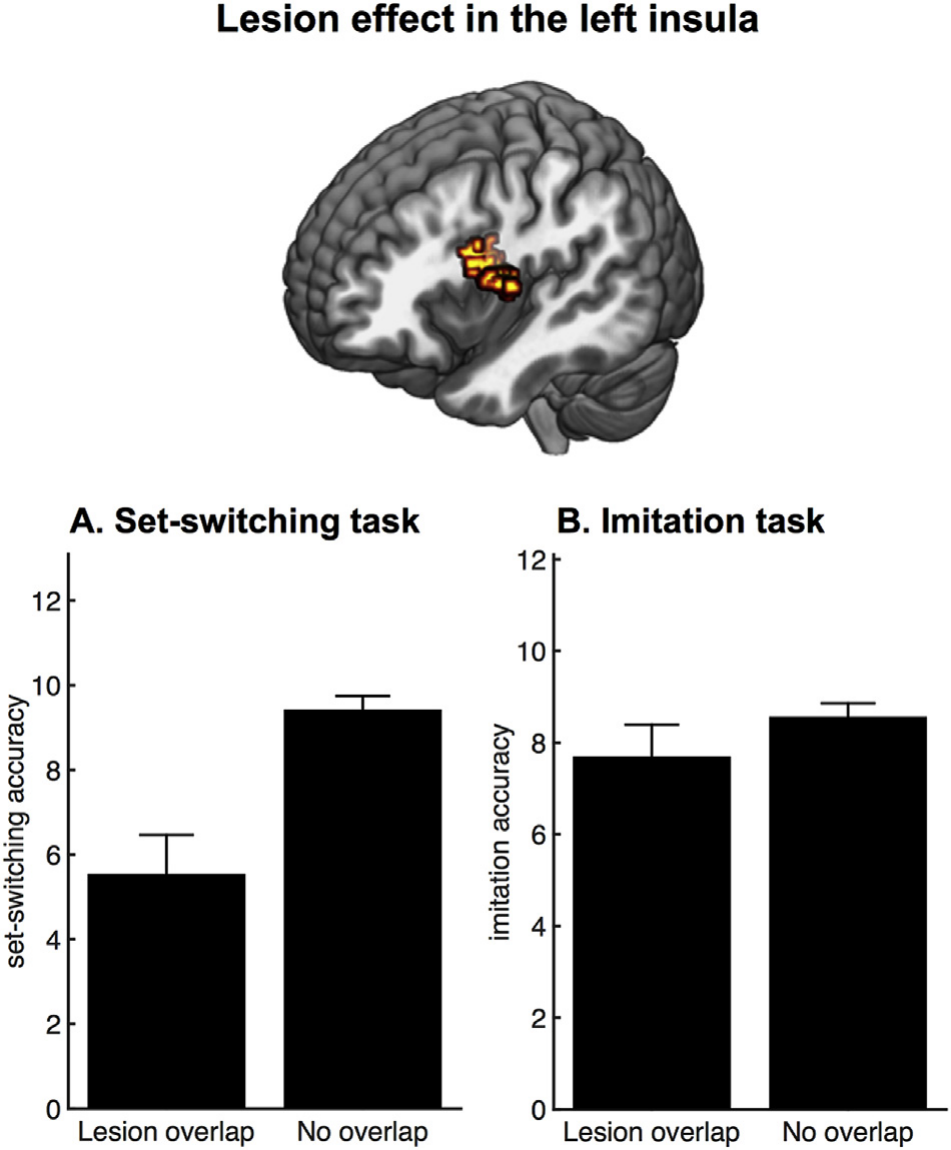
Cross-task specificity of the left insular lesion effect identified in the control VLSM analysis. A. Bar plot illustrating a difference in set-switching accuracy between the patients showing overlap (N = 13) versus no overlap (N = 131) with the left insular cluster. B. Bar plot illustrating no significant difference in imitation accuracy between the two groups. Error bars represent SEM.

A control analysis was performed to demonstrate the cross-task specificity of the left insular lesion effect. According to a predefined lesion overlap threshold (10%) (see Methods, section 2.3.2.), the lesion of 13 out of the 144 patients overlapped with the left insular cluster (as depicted in Fig. 3B). We compared performance of the two patient groups (overlap/no overlap with the left insular cluster) on the set-switching test and an unrelated imitation task. Specifically, a mixed ANOVA with lesion location (overlap/no overlap with the left insula cluster) and task (set-switching/imitation) as factors showed a main effect of lesion location (F_1,142_ = 6.84, *p* = *.01*), a main effect of task (F_1,142_ = 5.46, *p* = *.02*), and a significant interaction between lesion location and task (F_1,142_ = 6.68, *p* = *.01*). Post-hoc independent samples *t*-tests confirmed that there was a significant difference between the patient groups (overlap/no overlap) in the set-switching test (t_142_ = 3.55, *p* = *.001*), but not in the imitation task (t_142_ = .88, *p* = *.38*). In summary, a lesion in the left insula discriminated between high and low accuracy scores on the set-shifting test (Fig. 4A), while it was not discriminatory of high and low accuracy scores on an imitation task (Fig. 4B).

## 4 Discussion

This study used voxel-based lesion-symptom mapping (VLSM) in a large sample of acute stroke patients to identify regions critical for mediating flexible switching of attention. We used a shape-based analogue (Demeyere et al., 2015) of the well-established (Sánchez-Cubillo et al., 2009) and validated (e.g., Arbuthnott & Frank, 2000) metric of executive set-switching i.e., the Trail Making Test (TMT). The data showed that patients with localised damage to the left insular cortex performed worse in the set-switching test of the shape-based TMT analogue compared to patients without such damage. The finding was corroborated by further control analyses which demonstrated that the negative impact of left insular damage on executive set-switching (i) survived correction for the non-executive components of the task, (ii) was replicated in the VLSM analysis of the baseline-corrected executive scores (Demeyere et al., 2015); (iii) was replicated in the analyses restricted to patients with unilateral lesions; and (iv) was replicated in the analysis restricted to right-handed patients. Categorisation of patients based on the extent of their left insular lesion overlap further illustrated the specificity of the lesion effect to the executive demands of the shape-based TMT analogue. Specifically, the left insular damage discriminated between high and low accuracy scores in the set-switching test, but not in the baseline tests or in the imitation task, which matched the set-switching test in terms of difficulty level, though measuring a separate cognitive domain (praxis). This finding reinforces the notion that the lesion effect in left insula was not driven by patients' inability to comprehend the task instructions, overall poor task performance, or the ability to retain task-relevant information in short-term memory. Overall, this study provides lesion-based evidence supporting a role for the left insular cortex in executive set-switching above and beyond low-level visuospatial and motor components of the test.

The current study used a shape-based TMT analogue that differs from the standard TMT in a number of ways. Firstly, the shape-based TMT variant utilises shape-based stimulus sets, assessing executive deficits independently of number and letter sequencing impairments (Demeyere et al., 2015). Secondly, the task involves the sequential administration of two baseline tests, in which participants are required to connect task-relevant shape stimuli that are embedded in an array of task-irrelevant distractor shapes. Thirdly, in contrast to the standard TMT protocol (e.g., Bowie & Harvey, 2006), speed is not emphasised by the test administration protocol. Finally, while performance accuracy is not the main outcome variable in the standard TMT (Bowie & Harvey, 2006), it is a commonly used metric to supplement the completion time measurement (e.g., Kopp et al., 2015, Stuss et al., 2001). Accuracy measurement may be particularly informative for describing aberrant executive functioning in patient populations that tend to exhibit highly variable completion time profiles. Given the differences between the standard and shape-based TMT variants, the extent of generalizability of the findings obtained with the latter should be evaluated in the future studies.

Previous VLSM findings of executive deficits in brain-injured samples have implicated diverse frontal regions in TMT set-switching, including anterior cingulate cortex (Gläscher et al., 2012), right dorsolateral prefrontal cortex (Kopp et al., 2015), left dorsomedial prefrontal cortex (Miskin et al., 2016) as well as regionally non-specific left-lateralised prefrontal cortex (Barbey et al., 2012). A number of methodological aspects might have contributed to the variability of the reported frontal regions in these studies, as well as inconsistencies with the current study. In particular, across the above-described VLSM studies, lesion effects were identified in relation to various TMT-derived measurements that differed both with respect to the metric used (e.g., completion time versus accuracy) and with respect to the type of baseline correction. For instance, a VLSM study of chronic brain-injured patients used standardised, covariate-corrected and baseline-corrected completion time residuals as the outcome variable (Gläscher et al., 2012). Another VLSM study of war veterans with penetrating, cortical lesions used baseline-corrected completion time scores recorded in the number-letter switching condition from the D-KEFS variant of the TMT (Barbey et al., 2012). Finally, a study of acute stroke patients with right-lateralised lesions used both completion time and accuracy metrics, but identified significant lesion effects only in relation to the latter (Kopp et al., 2015). It should also be noted that the above VLSM findings of executive set-switching were based on aetiology-diverse chronic samples (Gläscher et al., 2012, Miskin et al., 2016), some of which included a stroke subset (Gläscher et al., 2012). In this study, we analysed behavioural and imaging data collected in a large stroke cohort within 3 weeks (average 5 days) post-injury. Importantly, the use of acute stroke sample minimised the contribution of remote, non-damaged brain areas that may support executive functioning during the chronic phase of stroke as a result of functional brain reorganisation (e.g., Carter et al., 2012, Pascual-Leone et al., 2005).

The lesion distribution of the current stroke sample fits with the empirical observation that the majority of the lesions induced by stroke affect subcortical structures (Corbetta et al., 2015). More specifically, empirical reports suggest that fewer than 20% of total stroke cases include lesions to cortical locations (e.g., Corbetta et al., 2015). By extension, the current sample exhibited a relatively low cortical lesion overlap in frontal areas and this, combined with a stringent minimum voxel overlap criterion (N = 10), may have limited the sensitivity of the VLSM analysis to detect significant effects in these locations. The negative result concerning the frontal regions therefore should be interpreted with caution. Another important methodological consideration of this study is that the patients with egocentric visual neglect were screened out. The right-lateralised lesion topography commonly identified in relation to the visuo-spatial neglect syndrome (e.g., Karnath and Rorden, 2012, Verdon et al., 2010), implies that the sensitivity of the VLSM analysis to detect effects in the right hemisphere might have been reduced. Whereas the purpose of such restrictive sampling was to minimise the contributions of specific visuospatial deficits to the executive set-switching measurement, accumulating evidence suggests a modulatory relationship between visual and executive aspects of attention in stroke (e.g., Singh-Curry & Husain, 2009). More research is therefore warranted to characterise the co-occurrence of executive impairments and hemi-spatial visual neglect in acute and chronic stroke cohorts, and to further elucidate to what extent damage to regions previously identified in association with specific spatial neglect components (Verdon et al., 2010) may underlie such a cross-domain relationship.

A characterisation of the left insular region identified in this VLSM study is warranted. While it is well-known that the insular cortex involves anterior and posterior subdivisions (e.g., Naidich et al., 2004), a probabilistic atlas reflecting a fine-grained gyrus organisation of this region has only been recently published (Faillenot, Heckemann, Frot, & Hammers, 2017). A qualitative comparison between the left insular region isolated in this study and the insular micro-anatomical subdivisions (Faillenot et al., 2017), suggested that the micro-anatomy of the left insular cluster included both anterior and posterior components. It should be noted that the probabilistic map of insula is representative of a healthy, young population, while the identified left insular cluster here was based on an elderly cohort that suffered an acute brain injury. In addition, while there is consensus in the fMRI literature in support of the functional dissociation between anterior and posterior insula (e.g., Menon & Uddin, 2010), stroke lesions reflect the distribution of the underlying vasculature and this frequently obscures the boundaries of the functional areas. By extension, the lesion effect in the left insula reflects the resolution of the vascular territory affected in the current sample and therefore does not permit making fine-grained anatomical or functional distinctions of this region. A potential drawback of the current study is that the lesions were delineated on the clinical scans acquired in CT and MR modalities. Although we used age-specific templates that are appropriate for studies with mixed CT/MR modalities (Rorden et al., 2012), we cannot exclude that the heterogeneity in the spatial resolution of the scans may have influenced the accuracy of the lesion delineation.

Although executive deficits have not been commonly reported following insular lesions (Ibañez, Gleichgerrcht, & Manes, 2010), a recent single-case study found that brain damage caused by the left insular stroke led to an isolated executive impairment (Markostamou, Rudolf, Tsiptsios, & Kosmidis, 2015). In particular, the stroke patient from this study performed below a cut-off on all indices of executive functioning, including a metric of mental flexibility, but had no language, perceptual or memory impairments. Another combined, multi-centre study that mapped TMT set-switching performance on separate indices of grey matter and white matter damage, yielded complementary data for the left insular involvement in executive functioning (Muir et al., 2015). Specifically, the study found that ischemic stroke within the left superior longitudinal fasciculus, a white matter tract traversing peri-insular region, predicted TMT set-switching deficits in both acute and chronic stroke cohorts. Conversely, stroke involvement with the grey matter regions within an “executive network” (including lateral and medial prefrontal cortex, lateral parietal cortex and thalamus) was not associated with TMT set-switching deficits. Based on these data and consistent with the insular lesion effect identified in the current study, which included both grey matter and white matter compartments, it could be argued that white matter pathways traversing the insular region might be as important as insular grey matter for accurate set-switching.

More broadly, the current data fits with the recent network models of insular function that implicate this region in the task-dependent control of goal-directed behaviour (Menon & Uddin, 2010), in addition to its well-established role in visceral and sensory processing (Craig, 2009). For instance, a functional connectivity study anchored the anterior insula within a ‘cognitive control network’ due to its involvement during a cognitive-control task combining working memory and target-switching demands (Cole & Schneider, 2007). In addition, the anterior insula has been designated a core node within a “saliency network” due to its role in tagging salient environmental stimuli for additional, controlled processing (Menon & Uddin, 2010). These models provide a good framework for the task-based fMRI data indicating that executive set-switching operations rely on a large-scale distributed network of brain regions, extending beyond prefrontal cortex and including anterior insula. Indeed, two meta-analyses of neuroimaging studies that used switching paradigms identified anterior insula as consistently being activated across the switching studies (Derrfuss et al., 2005, Wager et al., 2004). Specifically, activation in the anterior insula was detected in task contexts of uninstructed set-switching initiated based on trial-and-error learning (e.g., Monchi et al., 2004), as well as in tasks using explicit cues to induce switching (e.g., Dove et al., 2000, Dreher et al., 2002). More closely related to the current study however, is the finding that bilateral insular activation discriminated between set-switching and control conditions in the context of the fMRI-adapted TMT (Zakzanis et al., 2005).

## 5 Conclusions

To our knowledge, this is the first large sample study that investigated the mapping between executive deficits assessed through a stroke population optimised TMT tool and brain lesions delineated from acute stroke scans. The study yields lesion evidence for the involvement of the left insular cortex and adjacent white matter in flexible switching of attention. Importantly, we observed that this effect was independent of low-level visuospatial and motor demands of the shape-based TMT analogue, and could not be explained by overall poor task performance. Our findings are in accordance with recent network models of insular function, postulating a role for this region in regulatory aspects of goal-directed behaviour. Based on the network-level accounts of the insular function, there are most likely to be additional brain regions that are critical for accurate set-shifting performance but went undetected in the current study due to sample limitations. Furthermore, although the focus of the current study was on the regional structural damage arising from stroke, accumulating evidence suggests that remote dysfunction can occur in structurally intact regions connected to the area of lesion (e.g., Carter et al., 2012). Therefore, a possible avenue for future research may involve the study of the neural mechanisms through which focal brain damage in insular cortex leads to alterations in functionally-connected brain regions.

## Appendix A Supplementary data

mmc1
