# Supplementary material for: The role of left insula in executive set-switching: Lesion evidence from an acute stroke cohort
Source: Cortex. 2018 Oct;107:92–101. doi: 10.1016/j.cortex.2017.11.009 (PMC6181803; doi:10.1016/j.cortex.2017.11.009)
Supplement: mmc1 [file mmc1.pdf]

The role of left insula in executive set-switching: Lesion evidence from an acute stroke cohort

Andreja Varjačić, Dante Mantini, Jacob Levenstein, Elitsa D. Slavkova, Nele Demeyere*, Céline R. Gillebert*

** equal contribution*

SUPPLEMENTARY FIGURES

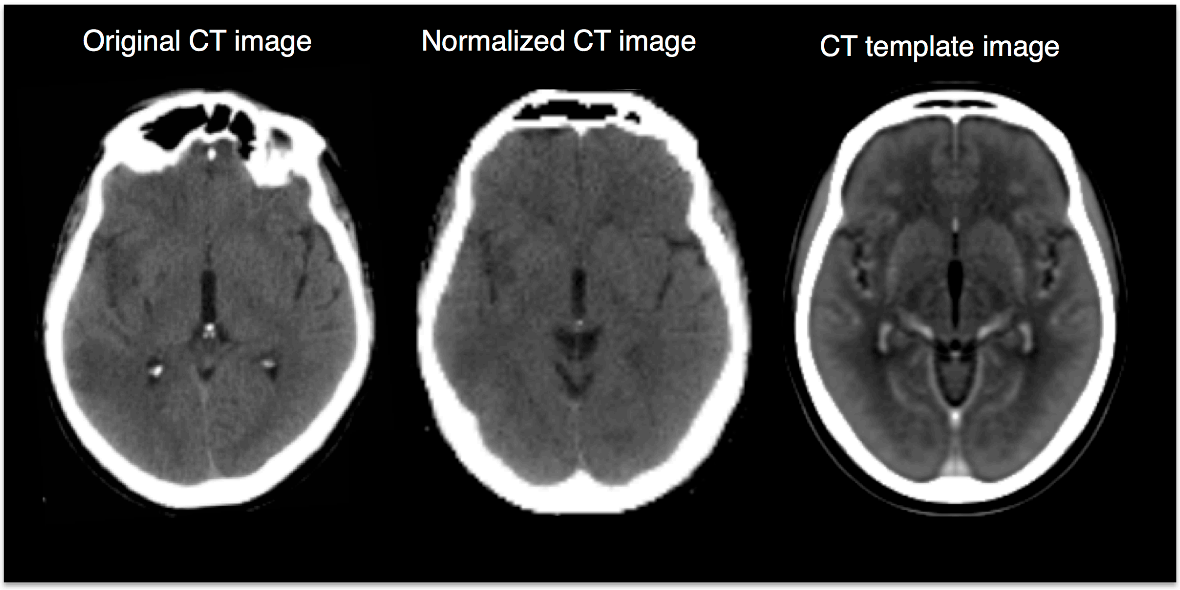

Figure S1. An example of the quality of the normalisation of a CT scans in a patient with damage extending into the left insular cortex.

VLSM of set-switching accuracy

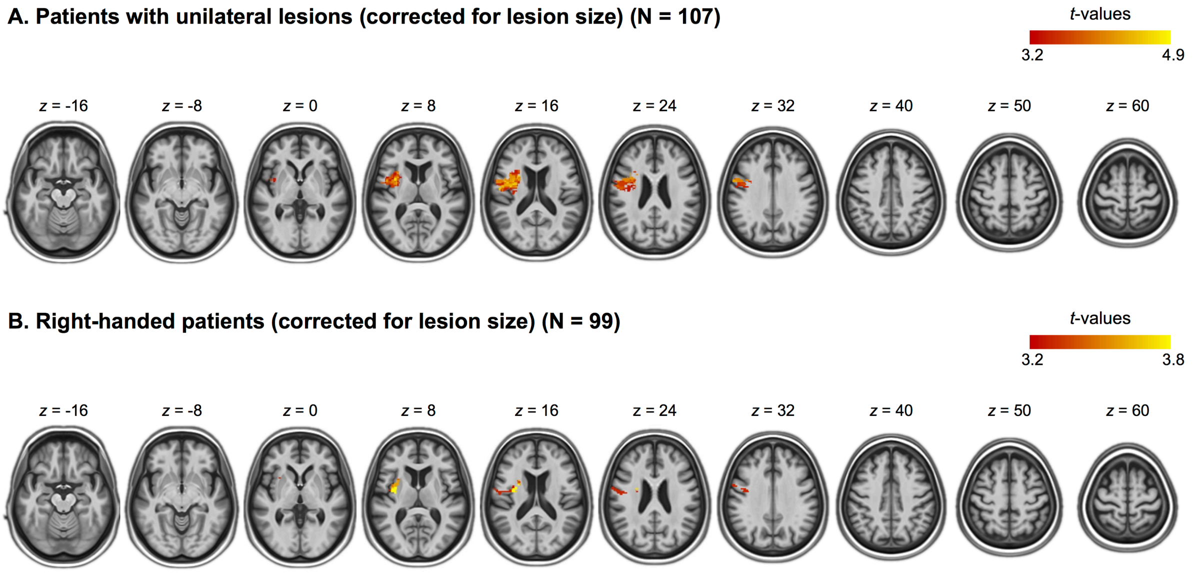

Figure S2. VLSM analysis of the set-switching accuracy in (A) the subset of patients with unilateral lesions (N = 107) and (B) the subset of right-handed patients (N = 99). In both analyses, lesion size was added as a covariate of no interest. For the analysis in the right-handed patients, voxels were included when lesioned in at least 5 patients. Images are displayed in neurological convention.

VLSM of executive scores

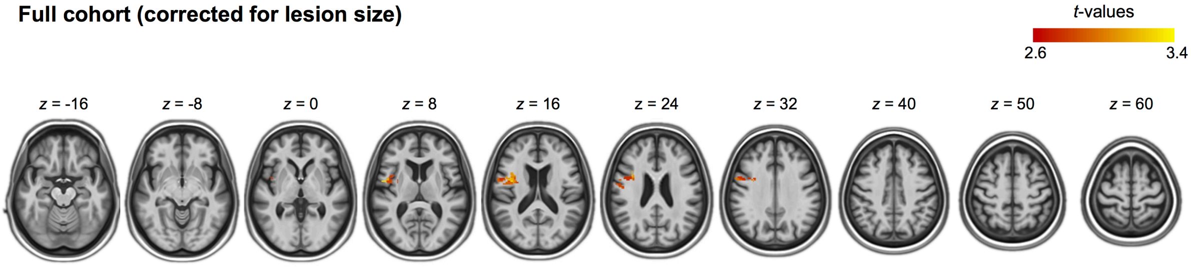

Figure S3. VLSM analysis of the executive scores, with lesion size as a covariate of no interest. Executive scores were expressed as a difference between summed baseline accuracy scores and set-switching accuracy scores. The displayed results survived a voxelwise threshold of *p<0.005,* corrected for multiple comparisons based on cluster size and the permutation method. Images are displayed in neurological convention.
